# Recent Advances in Colloidal Quantum Dots or Perovskite Quantum Dots as a Luminescent Downshifting Layer Embedded on Solar Cells

**DOI:** 10.3390/nano12060985

**Published:** 2022-03-16

**Authors:** Annada Sankar Sadhu, Yu-Ming Huang, Li-Yin Chen, Hao-Chung Kuo, Chien-Chung Lin

**Affiliations:** 1 Department of Photonics, Institute of Electro-Optical Engineering, College of Electrical and Computer Engineering, National Yang Ming Chiao Tung University, Hsinchu 30010, Taiwan; annadamcut@gmail.com (A.S.S.); s101328035@gmail.com (Y.-M.H.); hckuo@faculty.nctu.edu.tw (H.-C.K.); 2 International Ph.D. Program in Photonics (UST), College of Electrical and Computer Engineering, National Yang Ming Chiao Tung University, Hsinchu 30010, Taiwan; 3 Institute of Photonic System, National Yang Ming Chiao Tung University, Tainan 71150, Taiwan; chienchunglin@ntu.edu.tw; 4 Semiconductor Research Center, Hon Hai Research Institute, Taipei 11492, Taiwan; 5 Graduate Institute of Photonics and Optoelectronics, Department of Electrical Engineering, National Taiwan University, Taipei 10617, Taiwan

**Keywords:** QD LDS layer, solar cell, stability of QDs, solar cell with LDS layer

## Abstract

The solar cell has a poor spectral response in the UV region, which affects its power conversion efficiency (PCE). The utilization of a luminescent downshifting (LDS) layer has been suggested to improve the spectral response of the photovoltaics in the short wavelength region through photoluminescence (PL) conversion and antireflection effects, which then enhance the PCE of the solar cell. Recently, colloidal quantum dots (CQDs) or perovskite quantum dots (PQDs) have been gaining prime importance as an LDS material due to their eminent optical characteristics, such as their wide absorption band, adjustable visible emission, short PL lifetime, and near-unity quantum yields. However, the instability of QDs that occurs under certain air, heat, and moisture conditions limits its commercialization. Thus, in this review, we will focus on the physical and optical characteristics of QDs. Further, we will discuss different synthesis approaches and the stability issues of QDs. Different approaches to improve the stability of QDs will be discussed in detail alongside the recent breakthroughs in QD-based solar cells for various applications and their current challenges. We expect that this review will provide an effective gateway for researchers to fabricate LDS-layer-based solar cells.

## 1. Introduction

The rapid growth of the world’s population and the industrialization era have led to unprecedented energy consumption, which has resulted in environmental issues, concerns about environmental sustainability, and economic obstacles. Fossil fuels are currently utilized as an energy source for more than 80% of the world’s paramount energy and are directly involved in greenhouse gas emissions [1]. However, fossil fuels will not be able to fulfill the huge demand for energy in the future, due to their limited supply. Hence, there is a great need for the deployment of clean and renewable sources of energy. Renewable resources such as solar, hydraulic, biomass, wind, and geothermal energy have been researched and developed over the last few decades and are still under investigation. Among these, sunlight offers a prominent energy source, with an irradiation level of 1.8 × 10^14^ kW at the Earth’s surface [2], which can be converted into electricity and heat with a nominal impact on the environment [3]. Photovoltaic solar cells are among the most effective applications which convert optical energy (sunlight) to electrical energy. Therefore, with the invention of solar photovoltaic devices in the 1950s, numerous techniques and device designs have been suggested and achieved, such as single-/multijunction III-V solar cells [4,5,6], dye/quantum-dot-sensitized solar cells [7,8,9], chalcopyrite solar cells [10,11], and crystalline/amorphous Si solar cells [12]. In particular, photovoltaic (PV) panels based on silicon technology (crystalline silicon and polycrystalline silicon) are widely installed, owing to their eco-friendly and stable characteristics, superior PCE, and reliable supports in the semiconductor industry. Si-based solar cells have provided an outstanding efficiency of 25–26.7% in recent years [13,14,15]. On the other hand, GaAs-based III-V compound semiconductor solar cells have attracted huge attention due to their direct bandgap properties that exhibit high absorption over the entire visible portion of the solar spectrum. Therefore, GaAs-based solar cells are regarded as a promising technology for a high PCE. A GaAs solar cell with a single-crystal structure and thin-film crystal devices achieved high PCEs of 27.8% and 29.1%, respectively [16]. 

Despite the high PCEs, solar cells such as Si, GaAs, CIGS, and CdTe still exhibit poor spectral response in short-wavelength regions [17,18,19,20]. In addition, the surface recombination loss and surface Fresnel reflection are too high in these types of solar cells [21,22]. Fresnel reflection occurs due to the refractive index discrepancy between substrate–air interfaces. This discrepancy can be efficiently controlled by inserting an antireflective coating (ARC) between surface–air interfaces, which also offers a significant impact on enhancing the light utilization of the solar device [23,24,25]. Furthermore, to minimize the surface Fresnel reflection by using ARC, we must also employ the entire solar spectrum efficiently to increase the PCE, especially in the ultraviolet (UV) region. However, in the UV region, high-energy photons are easily absorbed by the material near to the surface of the solar cell, and the recombination loss of the generated electron–hole pair near to the surface is also high [26]. For this reason, a solar cell with the ordinary design cannot utilize the short-wavelength part of the solar spectrum effectively. This is a barrier to achieving a high PCE for any kind of solar cell. Fortunately, this issue can be solved by converting high-energy photons into lower-energy photons; this phenomenon is known as the luminescent downshifting (LDS) effect [27,28,29,30]. 

In 1990, Hovel et al. first demonstrated the advantages of utilizing the LDS effect for solar cell applications [31]. Subsequently, various kinds of work have been reported using quantum dots (QDs) as an LDS layer incorporated into different kinds of solar cells, such as silicon [32], GaAs [33], CdTe [20], organic [34], and CIGS [35] solar cells, to enhance the cell performance in the short-wavelength region, resulting in a higher PCE. The LDS materials absorb the light in the UV region and re-emit photons in the visible wavelength region, which can enter profoundly into the solar cell. Due to the fewer nonideal recombination centers in the bulk [26,36], more photons can be used to generate electron–hole pairs, which has a significant impact on increasing the external quantum efficiency (EQE) and generating a higher short-circuit current (J_sc_). However, the open-circuit voltage (V_OC_) and fill factor (FF) do not change significantly, and as a result, the electronic characteristics of the device resistance or the semiconducting material are not changed. Hence, due to the high current generation, the PCE can increase efficiently. 

The host materials embedded with luminescent dye also have a high impact on maximizing the benefits of the LDS layer and reducing additional losses. A suitable host material should have a high transmittance and a low scattering effect in the high-response region of the cell. Many researchers have reported different kinds of host materials, such as polyvinyl acetate (PVA) [37,38], polymethyl methacrylate (PMMA) [28,39], poly(dimethylsiloxane) (PDMS) [40,41], CaF_2_ [42], Al_2_O_3_ [31], and SiO_2_ [43]. 

A proper LDS material should contain some significant characteristics, such as (i) a wide absorption band, especially in the low-EQE region of the cell; (ii) a high absorption coefficient; (iii) a high luminescence quantum yield (LQE); (iv) a confined emission band that corresponds to the EQE peak of the cell; (v) a large Stokes shift, in order to decrease the losses due to reabsorption; (vi) high photostability; and (vii) low cost. 

There are essentially three types of LDS materials that have been reported so far: (i) rare-earth ions/complexes [37,38,42,44]; (ii) organic dyes [28,39,45,46,47]; and (iii) QDs [48,49,50,51,52]. Among these, QDs provide significant advantages over organic dyes and rare-earth complexes. QDs have a broad absorption band and a tunable emission band, a large Stokes shift, high photostability, and a high emission intensity [52,53,54]. On the other hand, rare-earth complexes have a high LQE [55,56] but a relatively low absorption coefficient [56]. Organic dyes have a small Stokes shift, a narrow absorption band [57], and poor photostability [31,58]. Semiconductor-based QDs, such as CdS, CdSe/ZnS, CsPbBr_3_, and CsPbI_3,_ have recently gained prime importance as the LDS materials of solar cells due to the great improvements to the PCEs of solar cells [33,41,59], whereas they may be instable when exposed to air, heat, and moisture [60,61]. Therefore, many works have been reported that focus on improving the stability issues of QDs, perfecting them for practical application. 

Thus, in this review, we will first discuss the physical and optical properties of QDs. Secondly, different approaches to the stability improvement and synthesis process of QDs will be presented. Thereafter, we will focus on the application of LDS layers in different kinds of solar cells. Finally, the challenges of the QD-based LDS layer for solar cells will be illustrated. 

## 2. Physical and Optical Characteristics of QDs

QDs are three-dimensional confined nanocrystals, and their electronic and photonic characteristics could depend on their size [62,63,64]. Due to the quantum confinement effect, the energy bandgap increases as the size of the QDs decreases. Hence, it is viable to obtain different emission colors by tuning the size of QDs. Colors such as violet, blue, and green are produced by smaller QDs, whereas yellow, orange, and red are produced by larger QDs. In view of their electronic wave functions and discrete electronic states, QDs are considered artificial atoms, since they are more akin to atoms than bulk materials [65,66]. Due to their small size, the electron of these nanoparticles is confined, and the energy levels are quantized according to Pauli’s exclusion principle when the nanocrystal radius is less than the exciton Bohr radius [67,68]. Their tunable emission, high thermal and optical stability, high quantum yield, and very short fluorescent lifetime make QDs promising candidates for application in field-effect transistors [69], photodetectors [70,71], solar cells [33,72,73], LEDs [74,75], biological fields [76,77], and visible-light communications [78]. Cd-based QDs have been one of the most prominent commercially available QDs used in optoelectronic applications for many years. However, Cd^2+^ ions are toxic in nature and have a negative impact on devices and tissue cells [79,80]. Consequently, scientists have concentrated their efforts on producing Cd-free light emitters that are as efficient, brilliant, and long-lasting as their environmentally harmful competitors. 

**Figure 1:**
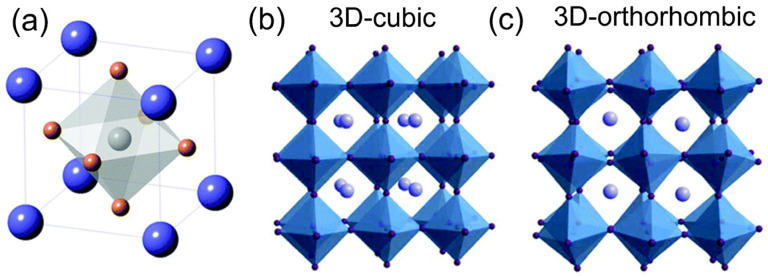
Perovskite structure in (**a**) crystalline state, (**b**) cubic state, and (**c**) orthorhombic state [85]. Figure reproduced with permission from John Wiley and Sons.

Perovskite QDs (PQDs) are a class of direct-bandgap semiconductor materials that feature a high photoluminescence quantum yield (PLQY) [81] and a high PL intensity and exhibit tunable narrow emission with symmetric PL peaks by altering the halide configuration [82]. Due to these unique properties, PQDs are emerging as the next-generation materials in photonic applications. In addition, being highly chemically processable and possessing superior optical properties, PQDs make excellent models of colloidal nanocrystals (NCs). PQDs can be blended efficiently using cost-effective precursors at ambient temperature or at a lower temperature (<180 °C) and exhibit both high electron mobility and a large exciton diffusion length [83,84]. PQDs are 3D-structure-based materials that can be expressed chemically as AMX_3_, where an organic cation (MA^+^ or FA^+^) or monovalent inorganic metal cations (Cs^+^ or Rb^+^) occupy the A-site; divalent metal cations (Ni^2+^, Cu^2+^, Eu^2+^, Sn^2+^, Pb^2+^, etc.) occupy the M-site; and the halide anion (Br^−^, Cl^−^, or I^−^) occupies the X-site [85]. Figure 1a indicates the perovskite crystal structure, where the X anion occupies the center face of the cube and the A and M cations occupy the vertexes and the center of the cube, respectively. Figure 1b depicts a regular octahedral structure that can be formed with this M cation (namely Pb^2+^) and the six bounded X anions, which can indicate an optimal 3D cube-shaped perovskite structure in an octahedral PbX_6_ arrangement. Nevertheless, when the octahedral PbX_6_ arrangement deforms from the 3D cubic shape, the perovskite formation changes to a less symmetric orthorhombic shape (Figure 1c).

**Figure 2:**
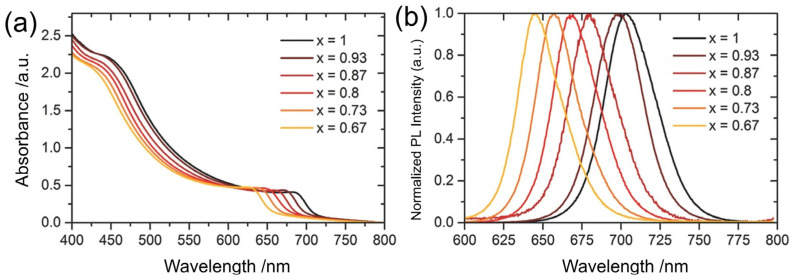
(**a**) Wide absorbance spectra with tunable absorbance peak near to UV region. (**b**) Tunable PL emission peaks in the visible-light region of the mixed halide CsPb(I_x_Br_1−x_)_3_ films with varying iodide concentration “x” for efficient LDS applications [92]. Figure reproduced with permission from John Wiley and Sons.

Perovskites are categorized into all-inorganic and hybrid (or inorganic/organic) materials. CsPbX_3_ belongs to the all-inorganic perovskite category, whereas MAPbX_3_ and FAPbX_3_ belong to the hybrid material category. Perovskite nanocrystals that are synthesized colloidally are typically categorized into QDs as 0D nanoparticles, nanowires (NWs), nanorods (NRs) as 1D nanoparticles, and nanoplatelets (NPLs) as a 2D nanocomposite. CsPbX_3_ crystals are very colorful. For instance, CsPbI_3_ crystals are black, CsPbBr_3_ crystals are orange, and CsPbCl_3_ crystals are pale yellow, while Cs_4_PbX_6_ (X = I, Cl, or Br) crystals have no color. In addition, CsPbX_3_ shows photoconductivity properties and exhibits maximal spectrum sensitivity towards violet (CsPbCl_3_), blue to green (CsPbBr_3_), and red (CsPbI_3_) [86,87]. Many researchers have reported that CsPbX_3_ perovskite is a good light absorber (Figure 2) due to its high absorption coefficient [88,89,90,91,92]. Despite having excellent physical and chemical characteristics, perovskite materials have been used in optoelectronics for just a few decades. The capability to fabricate colloidal PQDs in solution allows the production of cost-efficient emitting materials that are also suitable for flexible devices [93,94,95,96]. 

## 3. Synthesis Method of QDs

**Figure 3:**
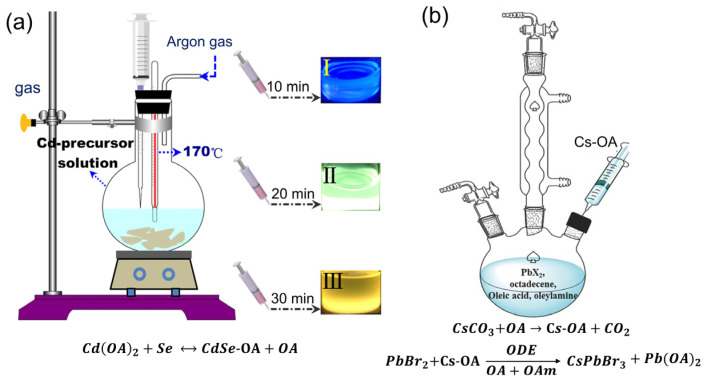
Synthesis process using hot-injection technique of (**a**) CdSe QDs ([97], figure reproduced under Creative Commons Attribution 3.0 license from IOP publishing) and (**b**) CsPbBr3 QDs ([83], figure reproduced with permission from John Wiley and Sons).

Recently, the most common method used for the production of QDs has been hot injection (HI). To produce QDs by hot injection, a pre-prepared precursor solution is injected into another while it is surrounded by a protective gas at a specific temperature. Due to the rapid nucleation and growth dynamics, this synthesis method can be completed in just a few seconds, resulting in QDs with good monodispersity, controllable composition, and a high PLQY. Figure 3a depicts the hot-injection synthesis process of CdSe QDs [97]. The materials needed to synthesis CdSe QDs are oleic acid (OA), cadmium oxide (CdO), octadecene (ODE), selenium powder (Se), and trioctylphosphine (TOP). By dissolving the selenium powder in TOP and injecting the Se–TOP solution into a hot Cd-precursor that comprises CdO, ODE, and OA, CdSe QDs can be obtained, as shown in Figure 3a. The inset of Figure 3a indicates that the emission colors of the QD range from blue to yellow under UV radiation, depending on the QD growth time.

Using ligands to cap QDs is one of the most well-known and simple processes for achieving a high PLQY. It is notable that the PLQY and optical characteristics of QDs are strongly associated with the surface ligands. The ligands play an important role in regulating the surface defects and passivating the dangling bond on the exposed surface of QDs, which subsequently modulate the deep trap states [98]. In this regard, Bansal et al. synthesized organic ligand-capped CdS QDs by the solvothermal synthesis method [99]. Tri-n-octylphosphine oxide (TOPO) and octylphosphine (TPO) were used as organic ligands, and a standard solvent for the precursor. A coordinating solvent, such as TOPO, and a noncoordinating solvent, such as ODE, OA, diphenylphosphine (DPP), or TOP, were used for the synthesis of CdS QDs. DPP/TOP acted as a sulfur ligand [98], and TOPO/OA acted as a cadmium ligand [100]. It is also notable that a high PLQY was only attained when OA was coupled with DPP to decrease the defects on both Cd and S atoms. Similarly, a high PLQY was also realized when TOPO/OA (or TOPO) was coupled with DPP (or TOP).

QDs synthesis in an aqueous solution is another effortless process. Nevertheless, this kind of water-soluble CdSe QD exhibits wide absorption and PL spectra but a poor PLQY. Through the hydrothermal process, it is possible to improve such poor optical properties of CdSe QDs. To prepare the precursor of CdSe QDs for the hydrothermal process, Cd ions and Se ions were mixed together with N-acetyl-L-cysteine (NAC) as a ligand [101]. The precursor solution was then treated to prepare the CdSe QD solution at a specific temperature using an oil bath for a particular period of time inside an autoclave and cooled in the ambient environment. CdSe/ZnS QDs were produced under a specific temperature of heating by constantly mixing the ZnS precursor solution into the CdSe QD solution.

Colloidal PQDs have also attracted huge attention in recent years due to their superior optoelectronic characteristics. They can be synthesized to achieve various goals such as a high PLQY, a cost-effective production, and a tunable bandgap. Colloidal PQDs have been synthesized by a variety of methods in recent decades [102,103,104,105,106]. HI process can also be used to synthesize colloidal PQDs. In 2015, Protesescu et al. first developed thoroughly inorganic CsPbX_3_ (X = Br, Cl, and I, or mixed halide) employing a cost-effective commercial precursor [107]. Figure 3b indicates the schematic picture of the synthesis procedure of CsPbBr_3_ PQDs using the HI method [83]. The Cs precursor solution contained CsCO_3_, ODE, and OA. The CsPbBr_3_ QDs were obtained by injecting the Cs precursor solution into a heated mixture of PbBr_2_, ODE, OA, and oleylamine (OAm).

**Figure 4:**
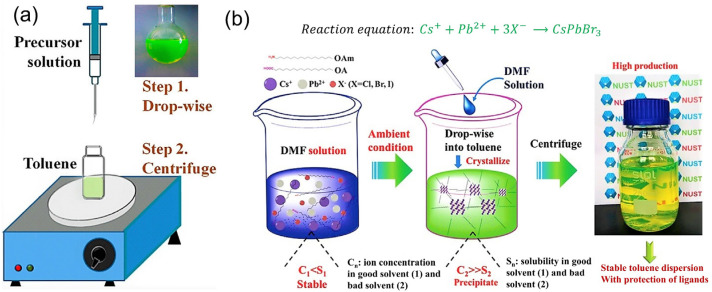
Synthesis steps of QDs using (**a**) LARP method ([110], figure reproduced with permission from ACS publications) and (**b**) RT-SR method with reaction mechanism ([111], figure reproduced with permission from John Wiley and Sons).

The HI process requires a specific injection temperature, a protective inert gas, and a high reaction temperature. Therefore, it is essential to develop a simple and easy-to-use technique to synthesize PQDs. The ligand-assisted reprecipitation (LARP) and supersaturated recrystallization (SR) techniques at room temperature have been reported in recent years. Based on the polarity of the reaction solvent system, two categories can be distinguished: nonpolar solvent systems and polar solvent systems. The reprecipitation technique is an easy procedure that employs solvent mixing to simultaneously produce polymer dots and organic nanoparticles. The usage of capping ligands on the surface of the nanoparticles has received a lot of attention recently, and it is becoming a more significant approach for regulating their size and configuration [108,109]. Zhang et al. proposed a LARP procedure to synthesize color-tunable and brightly luminescent colloidal CH_3_NH_3_PbX_3_ (X = Br, I, and Cl) QDs, which provided a PLQY of up to 70% at ambient atmosphere and low excitation fluencies [110]. Figure 4a schematically illustrates the fabrication of PQDs using the LARP process. The precursor solution for the synthesis of CH_3_NH_3_PbBr_3_ QDs was prepared by mixing PbBr_2_, CH_3_NH_3_Br, n-octylamine, and OA into a standard solvent, DMF, which has an excellent capability for dissolving inorganic salts and small molecules. The precursor solution was then gradually added into a vigorously stirred poor solvent, toluene, to eventually produce a yellow-green colloidal solution, indicating the aggregation process of the precursors into nanoparticles. Subsequently, small-sized nanoparticles were obtained by centrifuging the colloidal solution at a high rpm to remove large particles. Other longer chains of alkylamines, such as hexylamine, hexadecylamine, and dodecylamine, are also capable of controlling crystallization and producing colloidal QD solutions. However, it was found that n-octylamine had the potentiality to adjust the QD size by regulating the crystallization kinetics, while the aggregation of QDs was suppressed by OA, which assured the stability of the colloidal solution. Li et al. developed a room-temperature SR method (RT-SR) to fabricate CsPbX_3_ within a few seconds, without any inert gas or injection operation, as shown in Figure 4b [111]. A combination of ion sources containing CsX and PbX_2_ (X = Br, I) was mixed in DMF or dimethyl sulfoxide (DMSO) with the surface ligands OAm and OA to prepare a precursor solution. Subsequently, a supersaturated state was achieved due to the fast recrystallization after adding the precursor solution into toluene under vigorous stirring. Finally, after the centrifugation of the resultant solution, a stable toluene dispersion of PQDs with ligand protection was obtained (Figure 4b). Moreover, OAm and OA provided an advantage for tuning the crystal size and the ability to disperse them into various nonpolar solvents.

**Figure 5:**
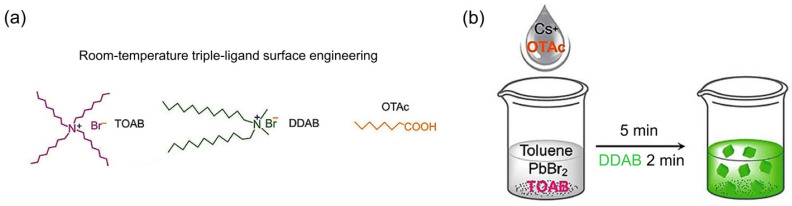
Schematic diagram of (**a**) room-temperature triple-ligand surface engineering and (**b**) synthesis and treatment procedure of PQDs [112]. Figure reproduced with permission from John Wiley and Sons.

Another work has been reported by Song et al. that focused on the synthesis of inorganic CsPbBr_3_ QDs using synergistic short ligands in toluene under room-temperature conditions without employing an inert gas as a protector [112]. Three synergistic short ligands, namely octanoic acid (OTAc), didodecyldimethylammonium bromide (DDAB), and tetraoctylammonium bromide (TOAB) (Figure 5a), provide excellent electrical transportation capabilities, a high ink stability and superior emissive properties, and a capability for dissolving PbBr_2_, respectively. Figure 5b describes the synthesis process of the PQDs.

**Figure 6:**
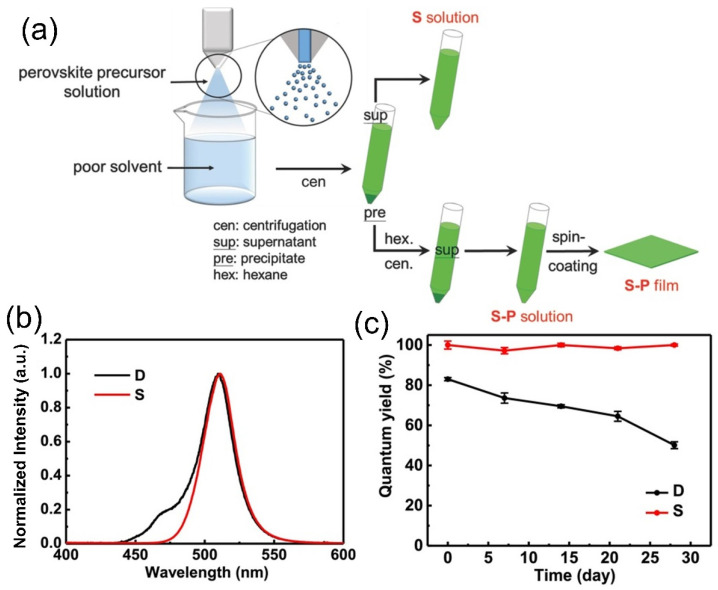
(**a**) Demonstration of spray-synthesis method; (**b**) PL emission spectra; and (**c**) stability performance of PLQY for the drop-synthesized (D) and spray-synthesized (S) solutions [116]. Figure reproduced with permission from John Wiley and Sons.

In most situations, the nanocrystallization of lead halide PQDs takes place by dispersing lead halides into standard solvents and then injecting them into poor solvents. Nevertheless, the PQDs generated using this method generally have a nonuniform spherical shape compared to the inorganic cesium (Cs)–lead halide analogs, which have a uniform cubic arrangement [113,114,115]. In addition, the PLQY of these PQDs is up to 80% in the solution form; however, this decreases to 40% in the thin-film form, due to the severe quenching effect [114]. To address this issue, Dai et al. developed PQDs using the spray-synthesis method by integrating conventional PQD synthesis with the spray-pyrolysis technique [116]. According to this work, both the solution and thin-film PQDs exhibited a uniform cubic structure with a stable PLQY of 100%. A standard solvent DMF was used to dissolve the organic halides, such as methylammonium bromide (CH_3_NH_3_Br, MABr), and lead bromide (PbBr_2_) with the organic ligands OA and n-octylamine, and this was sprayed into a poor solvent (toluene), as shown in Figure 6a. Unlike the conventional process, the micro-sized droplets in the spray enabled a significantly higher contact surface area and enhanced the mixing between the precursor solvent and the poor solvent, allowing for superior-quality PQD crystallization in the poor solvent. By centrifuging the resulting solution, the precipitate was eliminated and then put back into the hexane suspension to fabricate a spin-coated thin film. Figure 6b indicates the PL emission spectra of spray-synthesized QDs (S) and conventional drop-synthesized QDs (D) in toluene, where both QDs showed an emission peak around 511 nm. Nevertheless, solution D exhibited a shoulder peak at 470 nm due to the smaller size of the quantum dots in the solution. Figure 6c depicts the stability performance of the PLQY for solutions D and S, demonstrating that solution D was significantly less stable than solution S, with a high QY deterioration over 30 days. On the other hand, the PLQY of solution S was about 100%, but due to the presence of excess OA, it was burdensome to fabricate a uniform film. The casted film with excess OA experienced severe quenching, resulting in an exceptionally low PLQY of ~1–2%. In contrast, the film spin-coated with the precipitate of solution S in hexane revealed a very high PLQY of 100% and only 9% PLQY deterioration over 60 days. In conclusion, halide PQDs fabricated through spray synthesis exhibit excellent optical properties in terms of stability and emission when cast into thin films, suggesting that they are well-suited for photon conversion for optoelectronics devices.

The presence of Pb in lead halide PQDs results in toxicity issues, impeding their commercialization, even though they have a high PLQY. Researchers have reported various approaches for synthesizing Pb-free PQDs, such as substituting Pb^2+^ with Sn^2+^ [117] or partially substituting the B-site cation with various metal ions [118], which could maintain a standard PLQY and increase the thermal stability of α-CsPbI_3_ and orthorhombic CsPbBr_3_ at ambient environment. Thus, the concurrent exchange of the anion and cation in PQDs can improve the thermal stability as well as provide a high PLQY. Following this trend, Singh et al. developed a favorable approach by simultaneously exchanging the cation and anion with cobalt and chloride ions in the host CH_3_NH_3_Pb_1–x_Co_x_Br_3–2x_Cl_2x_ (x = 0 to 0.5) PQDs, followed by PMMA encapsulation for PQD stabilization [119]. The results indicated no crucial impact on the crystal structure after the partial substitution of Pb^2+^ and Br^−^ with Co^2+^ and Cl^−^, respectively. Moreover, after increasing the concentration of the Co^2+^ and Cl^−^ ion source, a blue shift in the PL was observed. This could be associated with the lattice contraction, along with the local anion clustering mechanism. It was also observed that after the partial substitution with Co^2+^ and Cl^−^, the bandgap of the PQDs changed from 2.29 to 2.41 eV, which was attributed to the electronegativity difference between the Pb^2+^ and Co^2+^ cations, as well as the Br^−^ and Cl^−^ anions. The host PQDs showed a PLQY of 95% after the substitution of the Co^2+^ and Cl^−^ ions.

**Figure 7:**
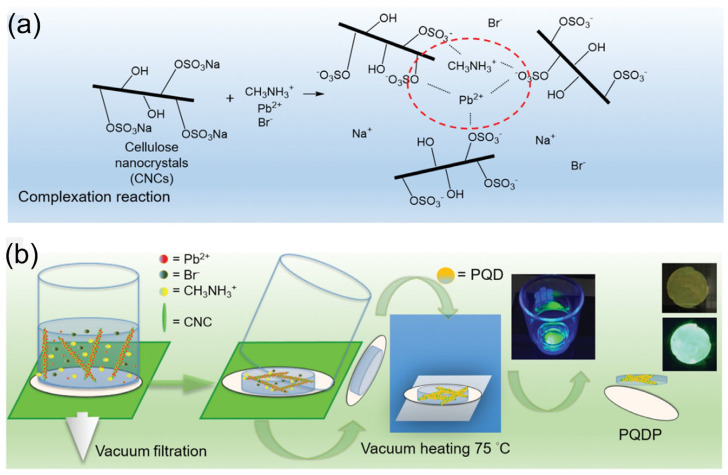
(**a**) Reaction mechanism between CNCs and anions in PQDs; (**b**) fabrication process of PQDPs based on oleic acid/oleylamine (traditional surface ligands)-free method [120]. Figure reproduced under Creative Commons CC BY license.

The use of surface ligands such as oleylamine (OLA) and OA during the synthesis stage is important for passivating the PQD structure, controlling the size of the PQDs, and stabilizing the PQDs in colloidal form. Additionally, encapsulation approaches have been utilized to boost both the moisture and the UV resistance of PQDs. Rather than incorporating carboxylic acids (such as OA) and alkylamines (such as OLA) in the perovskite precursor, Li et al. fabricated perovskite quantum dot papers (PQDPs) by casting cellulose nanocrystals (CNCs) as long-chain binding ligands, which contain a large number of -HSO_3_^−^ and -O^−^ groups (Figure 7) [120]. These two anions behave as capping ligands that restrict the growth of perovskite crystals and enable the forming of well-dispersed PQDPs. Moreover, during the preparation of the precursor solution (DMF + MAX + PbX_2_ + CNCs, MA = CH_3_NH_3_^+^) of the PQDs, the -HSO_3_^−^ and -O^−^ groups of the CNCs were coordinated to the empty orbital of the PbX_2_, and the PQDs were entangled between the long chains of the CNCs to avoid the dangling capping behavior of the OA and OLA and to increase the stability. The PQDPs exhibited a very stable PL intensity under continuous UV radiation for 60 days.

Table 1 demonstrates a comparison between Cd-based QDs and PQDs in terms of the improvement in the PLQY in recent years.

## 4. Strategies of Stability Improvement for PQDs

Despite its excellent physical and chemical properties, the perovskite material still has a lower stability compared with Cd-based QDs [122]. Furthermore, the low stability of hybrid organic–inorganic perovskite nanomaterials due to their organic component and their peripheral devices is a key issue for optoelectronic applications [123,124,125]. On comparison with organic–inorganic perovskite hybrid analogs, all-inorganic perovskite QDs such as CsPbI_3_, CsPbCl_3_, and CsPbBr_3_ have better thermal stability in the atmospheric environment [126]. Among them, CsPbI_3_ shows a comparatively confined bandgap (1.73 eV) at the cubic phase (α-CsPbI_3_), as well as better chemical and compositional stability [92,127,128]. However, a quick degradation occurs from its cubic phase (black phase) to a non-perovskite yellow phase (δ-CsPbI_3_, bandgap 2.82 eV) at room temperature [92,113,127,128,129,130], which then requires a very high temperature (above 310 °C) to change into the black phase from the yellow phase [127,128,130,131,132,133,134,135]. In this scenario, it is also necessary to note that the CsPbI_3_ black phase at room temperature is a cubic α-phase or orthorhombic γ-phase (bandgap 1.75 eV) [136,137,138,139]. Generally, both the α- and γ-phases have an optimal 3D perovskite structure in an octahedral PbX_6_ arrangement, whereas the non-perovskite yellow phase (δ-CsPbI_3_) has lost the 3D configuration [140]. Furthermore, both the α- and γ-phases show similar optoelectronics characteristics [137,139].

Thus, it is significantly difficult to employ CsPbI_3_ in a solar cell and a stable black phase at ambient temperature [141,142]. CsPbBr_3_ could be a substitute to overcome the black phase instability, but its large bandgap (2.25 eV) restricts photon harvesting, which then reduces the PCE of solar cells [143,144]. Therefore, many attempts have been reported to improve the phase stability of CsPbI_3_ in recent years. The most-often utilized processes are (1) 2D nanocrystal engineering, (2) doping with an alloying compound, and (3) solvent-additive techniques.

### 4.1. 2D Nanocrystal Engineering

CsPbI_3_ nanocrystals can improve the stability of CsPbI_3_ perovskite due to their immense surface energy and strong microstrain [107]. Nevertheless, the phase diagrams of size-dependent nanocrystals state that the size of the nanocrystals and the cubic phase stability of CsPbI_3_ are inversely correlated. Hence, α-CsPbI_3_ exhibits better stability as size of the nanocrystals decreases. Based on this hypothesis, Protesescu et al. demonstrated that cubic CsPbI_3_ 4–15 nm in size provided comparatively better stability when retained at room temperature for 30 days [107], while cubic CsPbI_3_ 100–200 nm in size strongly degraded into the yellow phase. Dutta et al. [145] reported that the phase stability of CsPbI_3_ also depends on the reaction temperature. This study then mentioned that colloidal and thermally stable CsPbI_3_ NCs can be produced via a reaction with high temperatures. The high temperature (~160 °C) allowed the alkylammonium ions to passivate the surface briskly and hinder the phase deterioration of the NCs. The attained NCs provided higher stability in the film both under an ambient environment and upon prolonged annealing at high temperatures.

Recently, Mir et al. [140] reported Mn-doped CsPbI_3_ NCs which showed better black phase stability for nearly a month compared to non-doped nanocrystals. The results revealed that the thermal and colloidal stability of the CsPbI_3_ black phase was improved due to the utilization of the postsynthesis Mn-doping procedure in an ambient environment via surface passivation. It could also be noticed that after the addition of the Mn dopant under ambient conditions into the CsPbI_3_ NCs, no structural changes occurred from the black phase (in both the α-CsPbI_3_ and γ-CsPbI_3_), and the transformation into the non-perovskite yellow phase (δ-CsPbI_3_) was prevented. Furthermore, these findings highlight the importance of addressing the fundamental chemistry of such NCs, which might assist scientists in improving the stability of nanocrystal-based inorganic perovskite solar cells.

### 4.2. Doping with Alloying Compound

**Figure 8:**
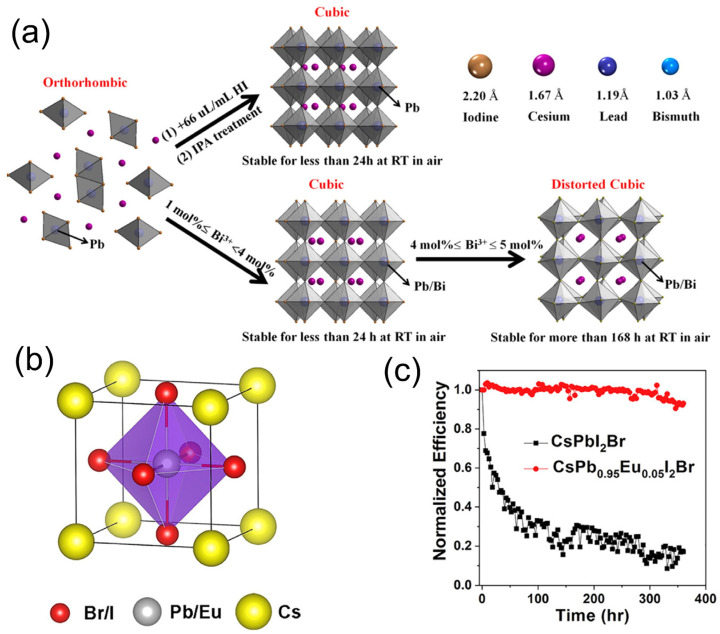
(**a**) Stabilization techniques of the α-CsPbI3 phase, utilizing hydroiodic acid (HI) or Bi^3+^ ([129], figure reproduced with permission from ACS publications); (**b**) crystal structure of Eu-doped CsPbI_2_Br; (**c**) comparison between Eu-doped and non-Eu-doped CsPbI_2_Br in terms of PCE stability ([149], Figure (**b**,**c**) reproduced with permission from Elsevier).

In order to increase the value of the Goldschmidt tolerance factor (t), the stabilization process of the CsPbI_3_ black phase can be carried out at ambient atmosphere by doping or partially alloying its B-site with metallic cations, which have a smaller radius [146]. As reported by previous works, the typical metallic cations used are Eu^3+^, Bi^3+^, Sb^3+^, Sn^3+^, Ge^3+^, and Mn^3+^ [147]. Hu et al. first proposed B-site doping using Bi^3+^ in CsPbI_3_ [129]. The 4 mol% Bi^3+^-integrated α-CsPb_0.96_Bi_0.04_I was produced by employing a one-step deposition technique and substituting Pb^2+^ (radius = 1.19 Å) with a Bi^3+^ cation (radius = 1.03 Å), resulting in a t-value increase from 0.81 to 0.84. The active layer of the solar cell with α-CsPb_0.96_Bi_0.04_I showed a high PCE of 13.21% and maintained 68% of the initial PCE for 168 h under atmospheric conditions. Moreover, it can be observed from Figure 8a that the stabilization techniques for the α-CsPbI3 phase are identical, whether hydroiodic acid (HI) or Bi^3+^ is utilized. The combination of Br and CsPbI_3_ to create CsPbI_2_Br has been shown to have high ambient stability and an appropriate bandgap (1.92 eV) for tandem solar cells [148]. However, in order to improve the stability further, Xiang et al. [149] recently proposed a CsPbI_2_Br perovskite solar cell doped with Eu^3+^ dopant (Figure 8b). This work shows a stabilized black phase and a significant improvement in the cell performance. In fact, after 370 h of uninterrupted white-light illumination at 100 mW/cm ^2^, the device exhibited 93% of the initial PCE, as shown in Figure 8c.

In 2017, another work developed by Akkerman et al. [150] improved the cubic phase stability of CsPbI_3_. In this work, an Mn^2+^ dopant combined with CsPbI_3_ was used to enhance the phase stability of the CsPbI_3_ film as well as its NCs. Nevertheless, it was also emphasized that due to the comparatively smaller radius of the Mn^2+^ cation (0.70 Å) with respect to Pb^2+^ (1.19 Å), only a small amount of Mn^2+^ could be used for the CsPbI_3_ NC phase separation. Due to the relatively close radii of Sn^2+^ (1.18 Å) and Pb^2+^ (1.19 Å), Sn^2+^ can be utilized to modify the t-value of CsPbI_3_. Nonetheless, due to the fast oxidation of Sn^2+^ in an atmospheric environment, it is necessary to incorporate it with another compound. Following this concept, Liang et al. [151] employed Br^−^ to synthesize a CsPb_1−x_SnI_3−x_Br_x_ mixed-halide perovskite, which provided superior phase stabilization.

**Figure 9:**
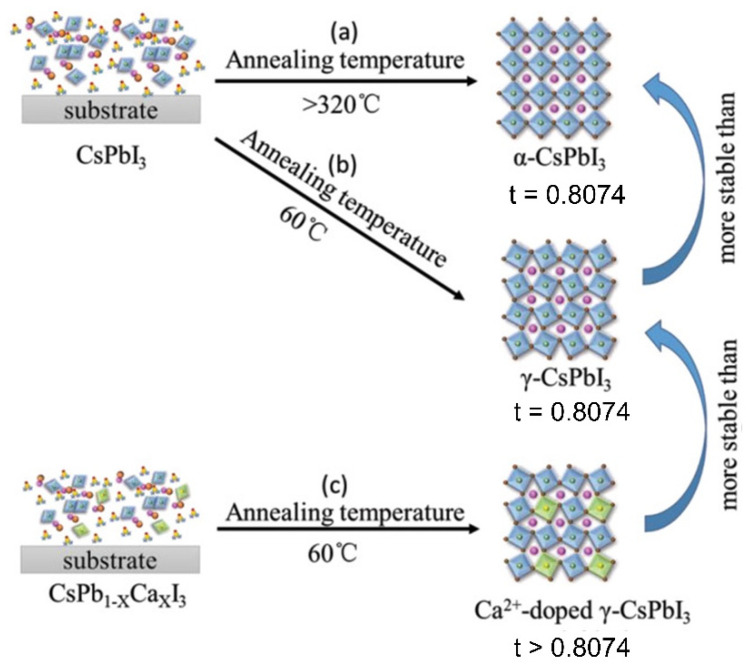
Formation and stability of (**a**) α-CsPbI_3_, (**b**) γ-CsPbI_3_, and (**c**) Ca^2+^-doped γ-CsPbI_3_ [138]. Figure reproduced with permission from John Wiley and Sons.

Recently, in 2020, Zhao et al. [138] reported that tortuous 3D γ-CsPbI_3_ films can be easily prepared under a low temperature (60 °C) without any additive and show similar optical properties to α-CsPbI_3_. However, this metastable tortuous 3D γ-CsPbI_3_ has a high tendency to convert into the non-perovskite yellow phase at ambient temperature. Ca^2+^-doped γ-CsPbI_3_ (CsPb_1−x_Ca_x_I_3_, x = 0–2%) has been found to provide a higher stability at room temperature. Figure 9 reveals that the formation of γ-CsPbI_3_ at 60 °C provides more stability than the formation of α-CsPbI_3_ (>320 °C), due to its lower cohesive energy. On the other hand, the formation of tortuous 3D γ-CsPbI_3_ doped with Ca^2+^ has better thermal stability than non-doped γ-CsPbI_3_, due to its lower cohesive energy and appropriate Goldschmidt tolerance factor (t).

Considering everything that has been said so far, it can be concluded that doping or alloying CsPbI_3_ with a small cation is a useful strategy to enhance the phase stability of the CsPbI_3_ layer. Nonetheless, further investigation is still required. Co-doping approaches might be an attractive route to pursue in further research.

### 4.3. Solvent-Additive Technique

In 2015, Eperon et al. first reported that the phase transformation of a CsPbI_3_ perovskite can occur at ambient atmosphere [127]. In fact, to form a black phase, it was necessary to rise the temperature above 335 °C. However, it could alter to the orthorhombic yellow phase when brought to an atmospheric environment. Nevertheless, a stabilized black phase with a 2.9% PCE could be produced at room temperature with the addition of a small amount of HI into the precursor solution (CsI and PbI at a ratio of 1:1 mixed in DMF) of CsPbI_3_. The purpose of adding HI was to produce microstrain in the crystal lattice, which caused the α-CsPbI_3_ to stabilize at room temperature. The entire process was performed in an inert environment at a temperature of about 100 °C, which was comparatively much lower than the initial temperature (above 310 °C) required to generate the black phase.

**Figure 10:**
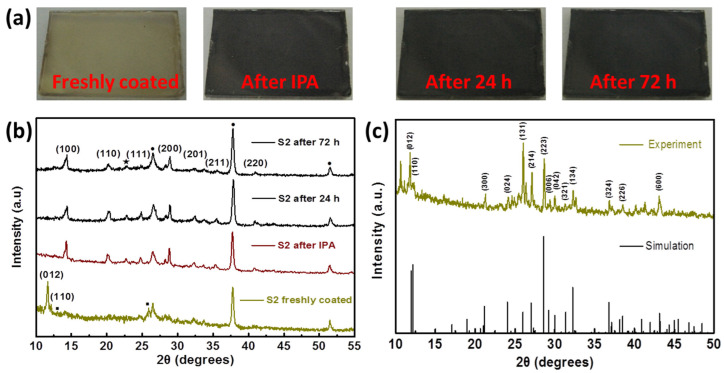
(**a**) The stable performance for 72 h of a freshly coated unstable film after treatment with HI and IPA; (**b**) their corresponding XRD patterns where star, black dot, and square represent the (112) plane of the orthorhombic (Pnma) lattice, the TiO_2_/FTO substrate, and PbI_2_, respectively; (**c**) experimental and simulated XRD patterns of the Cs_4_PbI_6_ phase [128]. Figure reproduced with permission from ACS publications.

In 2016, Luo et al. [128] demonstrated a sequential solvent process using HI and isopropyl alcohol (IPA) to fabricate a stable CsPbI_3_ at room temperature with a humidity of less than 30%. According to this work, the first 66 µL/mL of HI was mixed into the Cs_4_PbI_6_ precursor solution at a low temperature for optimization. After being mixed with HI, the film exhibited a yellow-brown color. Second, the film was annealed for 5 min at 100 °C after adding a hot IPA solution in atmospheric conditions. Eventually, the film converted to a dark brown α-CsPbI_3_ and provided a stability of up to 72 h, as shown in Figure 10.

**Figure 11:**
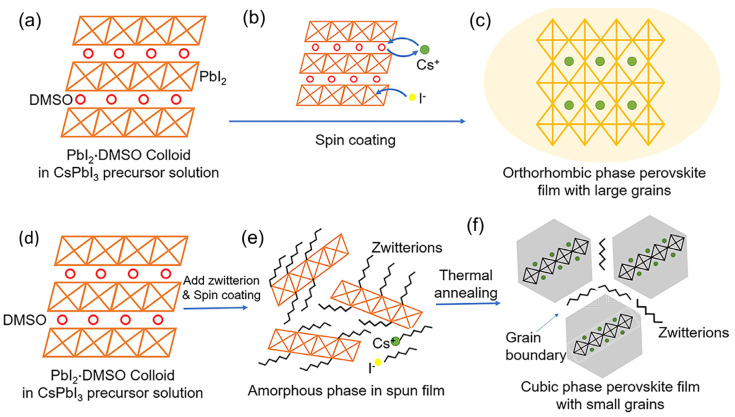
Mechanism of CsPbI_3_ *α*-phase stabilization by zwitterion: (**a**–**f**) schematic representation of CsPbI_3_ crystal formation from precursor solution without (**a**–**c**) or with (**d**–**f**) the zwitterion [155]. Figure reproduced with permission from Elsevier.

Recently, Zhao et al. proposed that the addition of a minute volume of H_2_O into the precursor solution of γ-CsPbI3 could provide thermodynamic stability to the γ-CsPbI_3_ films via a proton transfer reaction [139]. The theoretical and experimental results revealed that at surface areas greater than 8600 m^2^/mol, the γ-CsPbI_3_ film with lower surface free energy had better thermal stability compared to δ-CsPbI_3_. To increase the black-phase stability at ambient atmosphere, treatment with large-radius cations has been a found to be an alternative. For example, Li et al. [152] proposed that the addition of a large-radius phenylethylammonium (PEA^+^) cation into the CsPbI_3_ perovskite profoundly enhanced the phase stability of the film. In fact, the resulting Cs_x_PEA_1−x_PbI_3_ perovskite exhibited a PCE of 5.7% and α-phase stability at ambient temperature up to 250 °C in atmospheric conditions. Furthermore, Zhang et al. demonstrated that the incorporation of a small quantity of ethylenediamine cations (EDA^2+^) in the α-CsPbI_3_ perovskite showed superior black phase stability for a month at room temperature and more than 150 h at 100 °C, as well as a reproducible efficiency of 11.8% [153]. In 2016, Liao et al. introduced a 2D cesium lead iodide perovskite BA_2_CsPb_2_I_7_ (where BA denotes CH_3_(CH_2_)_3_NH_3_) with a combination of 3D CsPbI_3_ and a bulky ammonium cation. The synthesized BA_2_CsPb_2_I_7_ perovskite showed high stability under heating at 85 °C for 3 days and a relative humidity of 30% for one month [154]. Afterward, Wang et al. [155] used a single-step film deposition process to stabilize the α -CsPbI_3_ film in an ambient environment to incorporate a small amount of sulfobetaine zwitterions (~1.5 wt.%). By interacting with ions and colloids in the CsPbI_3_ precursor solution, the zwitterion molecules impeded the crystallization of the CsPbI_3_ perovskite. Under one-sun illumination, solar cells containing these zwitterion-stabilized perovskite films had a stable efficiency of 11.4%. Figure 11 describes the stabilization mechanism of the CsPbI_3_ α-phase using zwitterions.

The research reported thus far shows that incorporating additives into the perovskite manufacturing process not only improves the crystallization, but also stabilizes the material in its 3D crystal phase.

## 5. QDs with Various Solar Cells

One possible way to increase the photocurrent generated by a solar cell is to increase the number of photons collected by a device. The reduction in reflectance allows more photons to penetrate the devices, resulting in more carriers being produced and an increase in the photocurrent. A thin layer of QDs deposited on the surface of a solar cell can reduce the surface reflection due to its photon-scattering capability and intermediate refractive index (n = 2.4), which then increases the PCE of the cell [156]. Hence, QD layers not only have the ability of photon downconversion in the UV region but also offer an additional antireflection capability to enhance short-circuit current density, which further increases the efficiency of solar cells.

### 5.1. Si-Based Solar Cells

Si-based solar cells are currently the most widely used mainstream cell type for solar energy application, as they comprise 85–90% of the global PV module production. However, multicrystalline silicon (mc-Si) or polycrystalline silicon (p-Si) solar cells have a very low EQE at wavelengths below 500 nm, due to the low absorption and high reflection caused by the ARC, which is mainly optimized for only long wavelengths. They also exhibit an EQE of almost zero at wavelengths below 400 nm if the absorption of glass and encapsulants is increased [157]. Although crystalline silicon (c-Si) solar cells show a better performance up to a 400 nm wavelength, there is still a severe decrease in the performance at wavelengths <400 nm. On the other hand, amorphous silicon (a-Si) exhibits a major loss mechanism at short wavelengths due to the recombination of heavily doped semiconducting layers and the absorption in the transparent conductive oxide (TCO) layer. Thus, the utilizing of LDS layers can be a possible way to enhance the response at shorter wavelengths for silicon solar cells.

**Figure 12:**
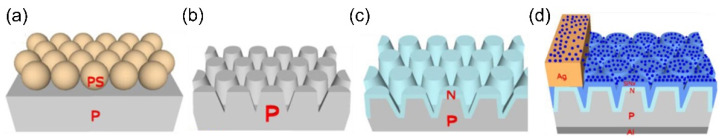
Fabrication steps of a c-Si solar cell containing NPAs incorporating a CdS QD/silica composite as an LDS layer: (**a**) spin-coat of closely packed polystyrene (PS) nanospheres monolayer on a p-type c-Si substrate; (**b**) resulting NPA after dry etching; (**c**) diffusion of an n-type layer indicates as N; (**d**) SiNx deposition and screen printing of the front and back electrodes, and then spin-coating of CdS QD layers on the device [32]. Figure reproduced under open access from Optica publishing group.

**Figure 13:**
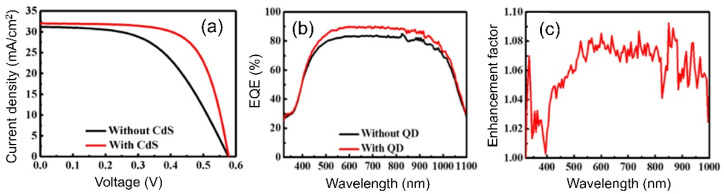
(**a**) Photovoltaic I–V characteristics of the c-Si nanopillar array (NPA) solar cell with and without CdS QD layers; (**b**) measurement of external quantum efficiency of the c-Si nanopillar array (NPA) solar cell with and without CdS QD layers; (**c**) peak (at ~335 nm wavelength) of short-wavelength enhancement in EQE, indicating photon downconversion [32]. Figure reproduced under open access from Optica publishing group.

A surface modification or coating of QDs to stabilize their photochemical and chemical characteristics is required to optimize the use of QDs for LDS layers of solar cells, since most LDS layers are on the top of the devices. Implanting QDs in a matrix as the protective shell can offer a high density of light-emitting centers. To support this hypothesis, Cheng et al. proposed a crystalline silicon (c-Si) solar cell that incorporated a CdS QD/silica composite as an LDS layer [43]. The cell without the LDS layer (pure glass) and the cell with the QD/silica composite were examined to compare their performances. The results showed that the J_sc_ of the cell with an adequate concertation of the QD/silica composite as the LDS layer increased up to 4% compared to that of the cell without LDS layer. In 2011, Chen et al. demonstrated a c-Si based solar cell with nanopillar arrays (NPAs) incorporating a CdS QD layer [32]. Figure 12 describes the fabrication steps of the proposed LDS-layer-based solar cell. The reactive ion etching and colloidal lithography processes were used to fabricate the NPA layer on the Si substrate. A passivation layer of silicon nitride was deposited after doping the surface with phosphorus oxychloride (POCl_3_) to form an n-type layer. CdS QDs were then spin-coated on the c-Si NPA as an LDS layer. For performance comparison, a cell without the CdS LDS layer was used as a reference. The J_sc_ of the cell with the QD layer was 32.03 mA/cm^2^ (Figure 13a), and the PCE increased from 9.45% to 12.45% (overall 33% improvement) compared to the reference cell. Besides this, a negligible change in the V_oc_ was observed. Moreover, the QD layer also decreased the series resistance of the cell, which directly improved the FF of the cell. Figure 13b,c clearly indicate the enhancement of the EQE in the visible-wavelength region due to the antireflection and scattering effect of the LDS layer. Additionally, the enhancement of the EQE in the short-wavelength region clearly demonstrates the photon downconversion capability of the QD LDS layer.

CdTe QDs embedded with a PMMA matrix were used by Kainarbay et al. to enhance the optical properties of polycrystalline silicon (p-Si) solar cells [158]. The spin-coated CdTe/PMMA composites with a thickness of 30 µm were deposited on the front surface of the device. Due to the wide absorbance band of CdTe (300 nm to 700 nm), it can absorb high-energy radiation and re-emit at a longer wavelength where the cell exhibits high spectral sensitivity. The cell with the CdTe/PMMA composites achieved an efficiency of 19.48%, which was much higher than that of the cell without the LDS layer (13.12%). The enhanced performance was attributed to the downconversion and antireflection effects of the LDS layer.

**Figure 14:**
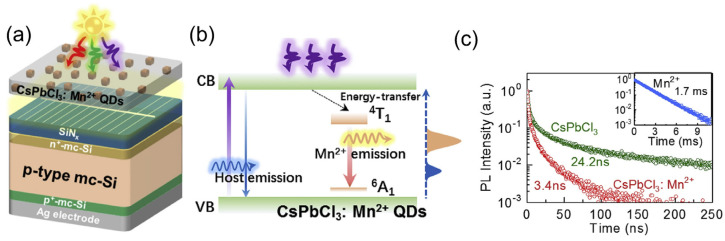
(**a**) Schematic diagram of a mc-Si solar cell with an LDS layer of CsPbCl_3_:Mn^2+^ QDs; (**b**) schematic of energy transfer mechanism of CsPbCl_3_:Mn^2+^ QDs in presence of excitation source; (**c**) lifetime and decay curve of CsPbCl_3_ and CsPbCl_3_:Mn^2+^ QDs [162]. Figure reproduced with permission from Elsevier.

**Figure 15:**
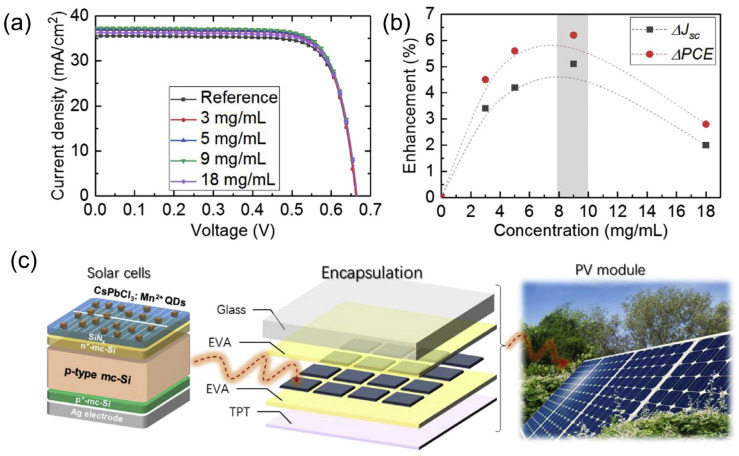
(**a**) JV curves; (**b**) ΔJ_sc_, ΔPCE of the mc-Si solar cells coated with various concentrations of CsPbCl_3_:Mn^2+^; (**c**) schematic diagram of a possible way for commercialization [162]. Figure reproduced with permission from Elsevier.

Halide perovskite nanocrystals are favorable emitters for cost-effective photonics and optoelectronics applications due to their easy solution-processed fabrication. Doping a certain number of metal ions, such as manganese [159,160], tin, zinc, and cadmium [161], into halide perovskite nanocrystals provides a level of independence in their configuration and allows them to entirely decouple their absorption and emission characteristics. To follow this hypothesis, Wang et al. demonstrated a p-type multicrystalline Si solar cell (mc-Si) device combined with an LDS layer of Mn^2+^-doped CsPbCl_3_ QDs on the front of the device to boost the spectral response as well as the stability of the proposed cell (Figure 14a) [162]. The 2.4% Mn^2+^-doped CsPbCl_3_ showed a PLQY of >60% and a large Stokes shift of more than 200 nm, with an emission peak at 602 nm. When the device absorbed UV light, the electrons were excited and reached the conduction band of the host material. Subsequently, the relaxation of the electrons occurred through the energy transfer to the ^4^T_1_ state of the Mn^2+^-ion (Figure 14b). Due to the opposite spin multiplicity of the ^6^A_1_ state and the ^4^T_1_ of the Mn^2+^-ion, the spin-forbidden ^6^A_1_-to-^4^T_1_ absorption transition has a negligible extinction coefficient [163], which resulted in a PL lifetime for CsPbCl_3_:Mn^2+^ of a few nanoseconds (Figure 14c). The CsPbCl_3_:Mn^2+^ QDs at an adequate concentration in the LDS layer efficiently enhanced the J_sc_ and PCE of the cell by 5.1% and 6.2%, respectively. However, when the concentration of CsPbCl_3_:Mn^2+^ QDs in the LDS layer was higher, the performance of the cell decreased compared with the cell without an LDS layer, due to optical losses (Figure 15a,b). Moreover, the devices with a CsPbCl_3_:Mn^2+^ QD LDS layer exhibited better performance in terms of photostability and reproducibility, which allows the possibility of further encapsulation processes for commercial applications (Figure 15c).

Table 2 indicates the benchmarks of recently reported silicon solar cells combined with an LDS layer of QDs from our group and other groups.

### 5.2. GaAs-Based Solar Cells

**Figure 16:**
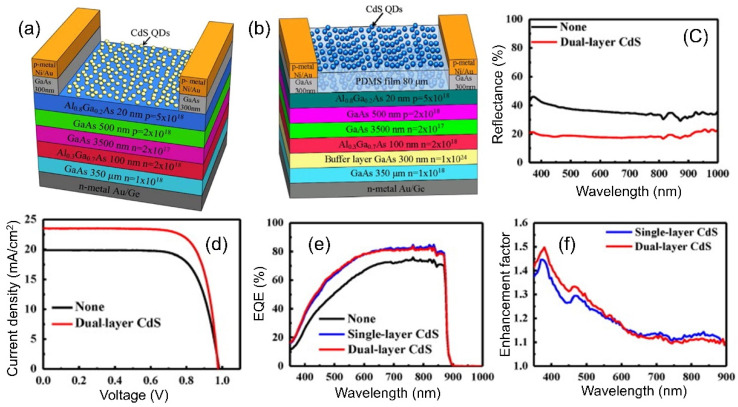
Schematic diagram of the fabrication steps of a single-junction GaAs solar cell combined with (**a**) a single layer of QDs without matrix [177] and (**b**) a dual layer of QDs with the bottom layer combined with a matrix. A comparative analysis of (**c**) reflectance spectra, (**d**) JV characteristics, (**e**) EQE, and (**f**) enhancement factor of a cell without QDs, with a single layer of QDs, and with a dual layer of QDs [40]. Figure (**a**) reproduced under open access from Optica publishing group. Figure (**b**–**f**) reproduced with permission from Elsevier.

GaAs is a promising candidate for solar-cell applications due to its direct bandgap characteristic, which exhibits strong absorption over the entire visible-wavelength region of the solar spectrum. In spite of the superior optical properties, GaAs solar cells show surface recombination losses and surface Fresnel reflection. LDS layers have been proposed to mitigate the issues encountered in GaAs solar cells. In 2012, Lin et al. demonstrated a highly efficient single-junction GaAs solar cell incorporating CdS QDs [177]. The single-junction solar cell (Figure 16a) was fabricated on a Si-doped n-type GaAs substrate via the subsequent deposition of several layers, chemical etching, and photolithography. CdS QDs (5 nm in diameter) were then deposited on the device as an LDS layer. The CdS QDs converted the photon from the UV region to the visible region. In addition, the QD LDS layer decreased the reflectance of the solar cell due to its intermediate refractive index (n ~ 2.4) compared with the cell without the LDS layer. As a result, an overall 18.09% enhancement in the PCE was observed in the solar cell incorporating the CdS QD LDS layer, nearly all of which was the result of the increase in the J_sc_ from 22.31 mA/cm^2^ to 26.99 mA/cm^2^.

Following this trend, a further report on QDs combined with a single-junction solar cell was published by Chen et al. [40]. In this work, the authors introduced a GaAs solar cell with a high PCE utilizing dual layers of CdS QDs. A spin-coated flexible PDMS film (~80 µm) was placed onto the first CdS QD layer that had been deposited on the surface of the device, and then the second CdS QD layer was coated onto the surface of the PDMS film through the pulse-spray coating (PSC) technique (Figure 16b). The PDMS layer served as an ARC and tuned the photon downconversion effect. On the other hand, the dual CdS layers provided excellent antireflective properties compared with the device without CdS QDs (Figure 16c), and they also absorbed the UV photon and converted it into multiple downconverted photons in the visible-wavelength region, which were efficient for generating the photocurrent in the bulk semiconductor. The results showed an enhancement in the J_sc_ from 19.87 to 23.52 mA/cm^2^ (Figure 16d) and an increase in the PCE from 14.36% to 17.45% (overall 22%), respectively, compared to the cell without an LDS layer. The change in the V_oc_ was negligible, but there was an increase in the FF as well as a decrease in the serial resistance, which could be attributed to the increase in the surface photoconductivity for the dual layers of the CdS QDs. On the other hand, the EQE of the cell with the dual CdS QD layer was almost 1.5-fold that of the cell without the dual layer (Figure 16e). It was also found that the solar cell with a dual layer of CdS QDs provided an extra 6% enhancement compared to the cell with a single layer of CdS QDs, as shown in Figure 16f.

In 2014, Han et al. developed highly efficient single-junction GaAs solar cells combined with three different kinds of QDs [33]. They used CdS (λ_em_ at 460 nm) and CdSe/Zns (λ_em_ at 530 nm and 640 nm) to check the quantum efficiency at different wavelength regions. The responses of the QDs deposited at different concentrations on the solar cells were recorded. The data showed that the green-emitting QDs, i.e., CdSe/ZnS, with an emission peak of 530 nm provided a more favorable response in terms of J_sc_, PCE, and EQE enhancement, respectively. The J_sc_ reached up to 22.87 mA/cm^2^, and the PCE was enhanced by up to 24.65% compared to the device without QDs. Furthermore, the V_oc_ and FF did not change significantly, which indicated that the QDs did not interfere with the diode performance. The improvement in the EQE and surface reflectance also suggested the photon downconversion and antireflection ability of QDs, respectively.

Hsu et al. analyzed the LDS effect and antireflection properties of a dual-junction GaAs solar cell incorporating QD layers in 2018 [59]. This work suggested that green-emitting QDs with an emission peak of 520 nm show better performance in terms of enhancing the J_sc_, PCE, and EQE compared to blue- and red-emitting QDs. The overall enhancement in the J_sc_ and PCE for the green-emitting-QD-embedded dual-junction solar cell were 9.37% and 5.47%, respectively, compared with a solar cell without QDs. A strong improvement in the EQE was also observed for the green-emitting-QD-embedded dual-junction solar cell, whereas there were no significant changes observed for blue- and red-emitting-QD-embedded devices. Nevertheless, a reduction in surface reflection was detected for all the QD samples due to their intermediate refractive index. Chen et al. also analyzed the LDS and antireflecting effect using CsPbBr_3_ PQDs with an emission spectrum of 530 nm deposited onto a commercially available single-junction solar cell [178]. Nanowhisker structures were introduced in this work to attach more QDs to the ITO without self-aggregating.

**Figure 17:**
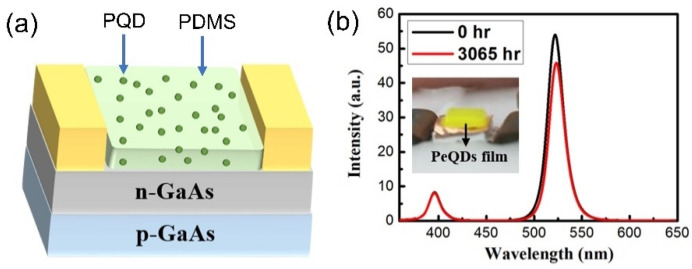
(**a**) Schematic diagram of the single-junction GaAs solar cell utilized CsPbBr_3_ PQDs embedded with PDMS; (**b**) stability performance after 3065 h [180]. Figure reproduced with permission from IEEE.

It is well known that PQDs usually suffer from low photostability and a low lifetime when exposed to moisture and heat [179]. In 2020, Huang et al. presented highly reliable and stable green-emitting (λ_em_ at 530 nm) CsPbBr_3_ PQDs incorporated onto a single-junction GaAs solar cell, which showed a long stability over 3065 h after fabrication [180]. The PQDs were mixed with PDMS at a weight ratio of 1:9 to form a gluelike solution, which provided high transmission. Subsequently, the gluelike solution was deposited on the top surface of the GaAs solar cell, as shown in Figure 17a. A SiO_2_ cladding layer was then used to coat the outside of the CsPbBr_3_ PQD surface to prevent degradation. Only a small wavelength shift was observed (~1 nm), which suggested that the good stability of the CsPbBr_3_ was protected from degradation by the SiO_2_ cladding layer (Figure 17b).

**Figure 18:**
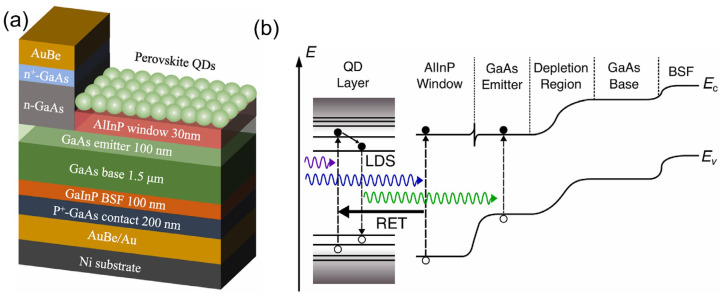
(**a**) Schematic diagram of the fabrication steps of a GaAs solar cell combined with PQDs on the top of the window layer; (**b**) photocurrent enhancement mechanisms [181]. Figure reproduced with permission from Elsevier.

To enhance the surface passivation, wide-bandgap semiconductors have been frequently utilized as the window layer of GaAs-based solar cells. However, such devices exhibit low photon-to-electron conversion efficiency in short-wavelength regions due to the parasitic absorption of the window layer. To improve the device efficiency, Rwaimi et al. proposed a GaAs solar cell that contained a window layer of AlInP and a FAPbBr3 PQD LDS layer [181]. The FAPbBr3 PQDs were spin-coated on the top of the window layer (Figure 18a) to enhance the EQE over its entire absorption band. Moreover, the QD LDS layer provided a large improvement in the short-wavelength region, resulting in a superior enhancement in the internal quantum efficiency (IQE), reduced surface recombination due to field-effect passivation, a well-matched refractive index, and an enhancement in the scattering. In addition, the window layer of AlInP improved the surface passivation, resulting in further enhancement in the IQE, which could be attributed to the nonradiative resonance energy transfer mechanism (RET) (Figure 18b).

Table 3 indicates the benchmarks of recently reported GaAs solar cells combined with a QD LDS layer from our group and other groups.

### 5.3. CdTe-Based Solar Cells

The CdTe-based solar cell would be considered as an eminent module for LDS application due to its spectral response, since a sharp cut-off in the spectral response at λ < 515 nm could be observed due to parasitic absorption in the CdS buffer layer. However, to improve the spectral response, the elimination or thickness reduction of these layers also results in a severe decrease in the efficiency [186]. It is possible to enhance the photon harvesting of the cell by blending the CdS buffer layer with zinc, which can allow more photons to be harvested into the cell by increasing the bandgap of the buffer layer [187]. Utilizing an LDS layer can be an alternative approach to collect more photons at a short wavelength. Hodgson et al. demonstrated a CdS_x_Se_1−x_/CdS/ZnS/PMMA-based LDS film incorporating a CdTe photovoltaic device to increase the photoresponse of the device in the short-wavelength region [20]. An enhancement in the EQE from 4% to 20% was observed in the 300–430 nm wavelength region, compared to the original device, while the LDS film was placed between the incident light and the device. However, at a longer wavelength, a loss in the EQE could be observed, which could be attributed to scattering loss, the primary absorption of solar radiation in the QD absorption tail, and the absorption and reflection caused by PMMA. It was also possible to increase the blue conversion by optimizing the thickness and concentration of the film and QDs, respectively, as well as by limiting the scattering and reabsorption. Another study from this group published in 2013 optimized the scattering losses due to PMMA and evaluated the LDS effect of the different concentrations of QDs on a CdS/CdTe solar device [188]. The QD/PMMA films provided high transparency at a longer wavelength of the visible-wavelength region. Furthermore, with increased QD concentrations, a small red-shift was observed in the fluorescence emission peak, which was attributed to a self-absorption effect. The J_sc_ and PCE of the device achieved improvements of 30.3% and 1.7%, respectively. As it stands, there are only a few studies that have been reported using a CdTe-based solar cell, which are listed in Table 4.

### 5.4. CIGS-Based Solar Cells

CIGS-based solar devices exhibit many similar properties to CdTe-based solar cells—in particular, short-wavelength absorption in the buffer layer, leading to a reduced EQE [190]. So far, only a few experiments have been reported that aimed to enhance the spectral response to short wavelengths of CIGS solar cells combined with a QD film as an LDS layer. However, based on the reported data, it is challenging to make conclusions regarding the optimal combination. In 2015, Laio et al. reported a flexible CIGS solar cell with a combination of self-assembled clusters of CdSe/ZnS core/shell nanocrystal QDs (NQDs) as an LDS layer [191]. The pulse-spray deposition technique was used to deposit a nonuniform layer of NQDs between the buffer layer and the window layer. An optimal pulse-deposition dose of NQDs can enhance the spectral response of the device in the short- and long-wavelength regions due to the LDS effect and the internal scattering of the nonuniform NQDs, respectively. A significant improvement in the J_sc_ from 31.9 mA cm^−2^ to 35.5 mA cm^−2^ (relative enhancement of 12.2%) and in the PCE from 8.42% to 9.34% (relative enhancement of 10.9%) were observed compared to the device without the LDS layer.

**Figure 19:**
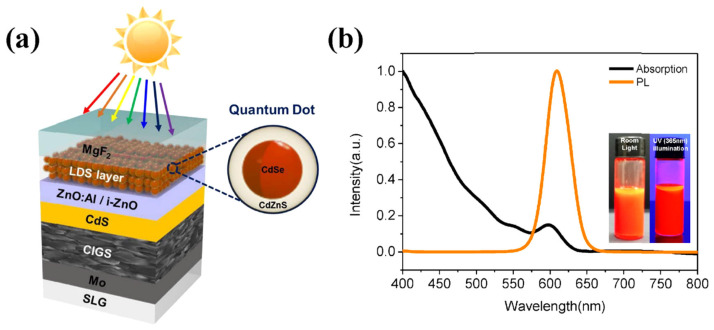
(**a**) Schematic diagram of the CIGS solar cell with both ARC and LDS layers; (**b**) absorption and PL spectra of CdSe/CdZnS QDs [192]. Figure reproduced with permission from ACS publications.

In 2017, Jeong et al. developed a CIGS solar cell incorporating an LDS layer of CdSe/CdZnS core/shell QDs to enhance the spectral response in the short wavelength regions [192]. The LDS layer, which was placed between the MgF_2_ ARC layer and the window layer (Figure 19a) enhanced the photon absorption in the short wavelength and re-emitted in the longer wavelength with a peak at 609 nm (Figure 19b). Subsequently, the re-emitted photon was absorbed in the CIGS layer, which was transmitted via the window and buffer layers of the cell. The EQE of the cell was increased by 51% in the 300–520 nm region and the PCE of the cell was increased from 13.76% to 14.29%, compared a cell without a QD LDS layer.

**Figure 20:**
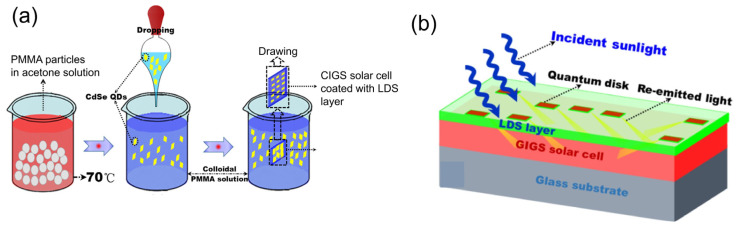
(**a**) Fabrication steps of QD/PMMA disks; (**b**) schematic diagram of the CIGS solar cell with an LDS layer of QD/PMMA disks [35]. Figure reproduced with permission from Elsevier.

In this same trend, CdSe quantum disks as an LDS layer of a CIGS solar cell were proposed by Li et al. in 2019 to improve the short-wavelength response of the cell [35]. The QD/PMMA disks were fabricated (Figure 20a) and applied on the top surface of the device (Figure 20b) to enhance the EQE in the range of 300-460 nm by up to 25%, and the output electrical power of the device was increased by up to 114%.

**Figure 21:**
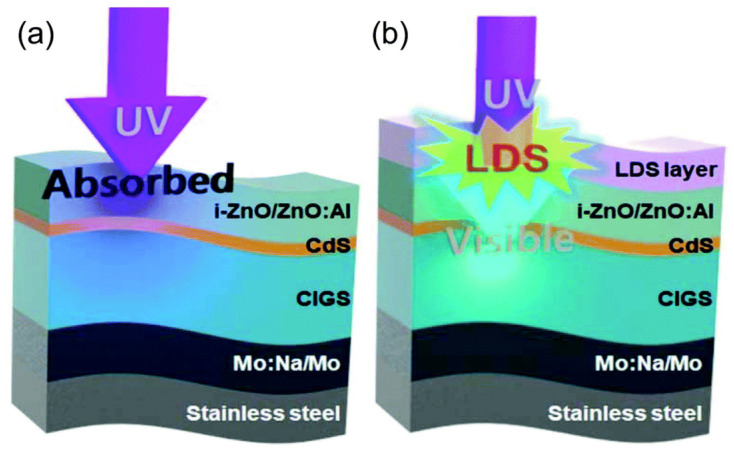
Schematic of the flexible CIGS solar cell (**a**) without LDS layer and (**b**) with the efficacy of LDS layer [193]. Figure reproduced under a Creative Commons Attribution Non-Commercial 3.0 Unported license from the Royal Society of Chemistry.

Most recently, Kim et al. developed a flexible CIGS solar cell integrated with an LDS layer of CsPbBr_3_ PQD NCs [193]. A stainless-steel foil with a thickness of 100 µm was used as a substrate to fabricate the proposed solar cell. A CsPbBr_3_ film with a specific thickness was spin-coated on the top of the device as an LDS layer (Figure 21b). The CsPbBr_3_ NCs enhanced the photon absorption in the short-wavelength region (<520 nm) by mitigating the parasitic absorption in the transparent conductive oxide (TCO) layer of the cell and re-emitted in the longer wavelength region (532 nm), which was further absorbed by the CIGS layer to generate the photocurrent. A huge enhancement in the EQE in the 300–390 nm wavelength region was observed. Moreover, the device also showed an enhancement in the EQE in the 500–1100 nm wavelength region due to the less significant refractive index mismatch between the air and the TCO layer due to the inserting of the LDS layer. As a consequence, both the J_sc_ and the PCE of the device improved by up to 4.5% compared to the cell without QDs.

Table 5 indicates the benchmarks of the recently reported CIGS solar cells combined with a QD LDS layer.

### 5.5. Organic and Perovskite-Based Solar Cells

In comparison to silicon solar cells, organic solar cells usually exhibit a shorter working lifetime as well as a low PCE, which is the main barrier to the commercialization of organic solar cells. Proper encapsulation with an efficient LDS material has been proposed to enhance the lifetime and efficiency performance of organic cells through the LDS effect. Aoki et al. developed a P3HT:PCBM organic photovoltaic cell encapsulated with an LDS layer of CdS/ZnS [34]. The HI method was used to prepare the QDs. The results showed that the organic cell with the LDS layer exhibited a significant improvement in the V_oc_, J_sc_, FF, and PCE compared to the reference cell without the LDS layer. Additionally, the stability of the organic solar cell with the LDS layer was much better than the device without the LDS layer.

**Figure 22:**
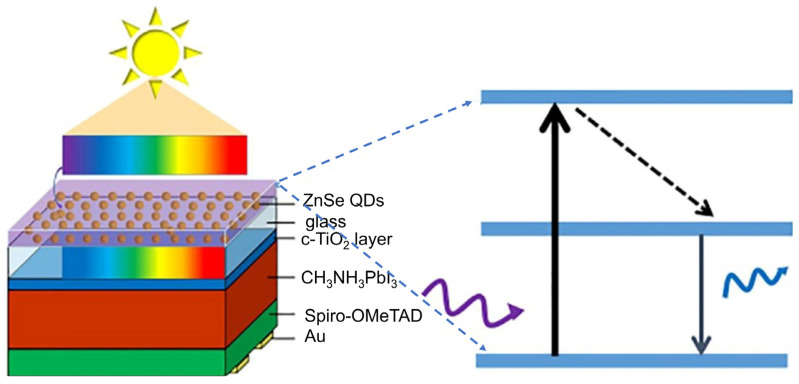
Schematic of PSCs with LDS layer of QDs, along with its working mechanism [196]. Figure reproduced with permission from Elsevier.

Perovskite solar cells (PSCs) have been gaining prime importance in recent years due to their unique properties, such as their superior charge mobility, good light-absorption capability, and simplistic fabrication technique [194,195]. However, the instability of PSCs is a key issue that has hindered their wide application and development. Both the humidity in the air and the UV rays from sunlight can significantly degrade the performance of PSCs. A suitable LDS material can absorb UV rays and transform them into visible light, preventing the degradation and increasing the light-harvesting of the perovskite layer. In 2017, Wang et al. developed an PSC that incorporated a spin-coated LDS layer of ZnSe QDs on the backside of the cell (Figure 22) [196]. The uniform size of the QDs was achieved using the HI method, which absorbed significant amounts of UV light and converted it into visible light. The emission spectra contained three different peaks at 400 nm, 435 nm, and 460 nm, which were attributed to the bandgap emission, surface trap state, and deep level state of the QDs, respectively [197]. The results revealed that the cell with QDs exhibited a PCE of 17.3% as well as better stability compared to the cell without QDs. In Section 4.2, we mentioned that alloying or doping CsPbI_3_ with smaller-radius metal cations can enhance the black phase stability of CsPbI_3_, which can be further used as an LDS layer to enhance the short-wavelength response for any kind of cell. In the same vein, Chen et al. developed highly stable PSCs which were integrated with air-stable Cs^3+^-CsPbI_3_ as an LDS layer to enhance the short-wavelength response of the cell [198]. The layer of Cs^3+^-CsPbI_3_ efficiently absorbed UV rays and converted them to light with a longer wavelength. The J_sc_ of the cell with the LDS layer was increased from 23.45 to 24.13 mA/cm^2^, and the PCE of the cell with the LDS layer was increased from 21.5% to 22.16%, compared to a cell without the LDS layer. In 2019, Chen et al. introduced the application of CsPbBr_3_ inorganic perovskite nanocrystals (IPNCs) as an LDS material inside of PSCs containing the structure of FTO/TiO_2_/IPNCs/CH_3_NH_3_PbI_3_/spiro-OMeTAD/Au [199]. A suitable interlayer of IPNCs was constructed via the spin-coating process, which acted as a seed-mediated layer to form a perovskite film with both an excellent absorption capability and a uniform morphology. Furthermore, CsPbBr_3_ and hybrid perovskite films provided a fully crystalline heterojunction which reduced the trap densities and defects. The device showed a red fluorescence due to the ion exchange of the I^−^ in CH_3_NH_3_PbI_3_ with the Br^−^ in CsPbBr_3_. The results revealed that at a certain thickness of CsPbBr_3_ (~110 nm), the PCE was enhanced from 14.7% to 16.4% (overall gain 11.6%) and the J_sc_ was enhanced from 22.49 mA/cm^2^ to 23.31 mA/cm^2^ (overall gain 3.64%), compared to a device without the IPNC layer inside the cell. After 100 h of UV radiation, the device with the IPNCs inside the cell exhibited high stability and retained 82% of its initial PCE, whereas the cell without CsPbBr_3_ lost 45% of its initial PCE. In addition, to check the LDS effect, air-stable CsPbBr_3_@SiO_2_ nanocomposites were deposited on the front side (transparent side) of the device. However, this device exhibited a PCE of 15.3%, which was lower than the CsPbBr_3_ inside of the device. The reason was that the IPNCs deposited on the outside surface only contributed the photon downshifting effect. After 100 h of UV radiation, the device with the IPNCs outside the cell showed high stability and only lost 6% of its initial PCE. This indicated that the cell with CsPbBr_3_ inside was less stable compared to the cell containing CsPbBr_3_ on the outside surface. This was due to the ion exchange occurring inside the device. Thus, to avoid the ion exchange inside the cell, CsPbBr_3_@SiO_2_ nanocomposites were deposited inside the cell. However, the PCE decreased to 6.7%. This was due to the dielectric nature of SiO_2_, which becomes a carrier-transporting barrier on the surface of IPNCs. the long-lasting performance was also measured using a bare cell that had a PCE of 19.7%. After coating the outside of the cell with CsPbBr_3_@SiO_2_ nanocomposites, the PCE of the device reached 20.8%, and the J_sc_ of the device was increased from 23.3 mA/cm^2^ to 24.6 mA/cm^2^ (4.53%). After 100 h of UV radiation, the device with CsPbBr_3_@SiO_2_ nanocomposites showed a high stability and retained 96% of its initial PCE, and after 1000 h of stimulated light irradiation, the device retained an average of 92% of its initial PCE. Therefore, the results showed that IPNCs acted perfectly as an LDS layer and improved the light response in the UV wavelength region when deposited either outside or inside the cell. Nevertheless, the IPNCs inside the cell showed a better PCE, due to the higher electrical conductivity and superior carrier transmission generated by IPNCs. Table 6 illustrates the benchmarks of recently reported organic and perovskite solar cells combined with an LDS layer of QDs.

## 6. Challenges of QD-Based LDS Layers in Solar Cells

**Figure 23:**
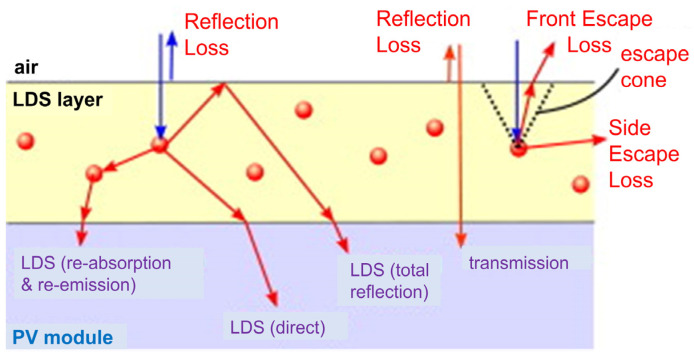
Schematic of different kinds of optical procedures and losses in the LDS layer [206]. Figure reproduced with permission from Elsevier.

An LDS layer applied to a photovoltaic device introduces more pathways for photons, which also introduces additional loss mechanisms. Hence, any possible improvement that occurs due to the shifting of the spectrum will need to mitigate these additional losses before calculating the overall gain. Both the luminescent material and the host material are responsible for the additional losses. The major physical phenomena that can occur include: (i) the host material exhibits parasitic absorption; (ii) the emissions from the luminescent material have a luminescent quantum efficiency (LQE) of less than l (the LQE is defined as the ratio of the number of photons emitted to the number of photons absorbed by a luminescent dye); (iii) reabsorption occurs because of the partial overlap of the absorption and emission spectra of luminescent species; (iv) light escapes, since not only the luminescent light that is emitted into the cell but also the losses through the top and side planes of the layer need to be considered; and (v) the reflection is enhanced due to the introduction of an additional interface. Figure 23 indicates a schematic diagram to represent different kinds of optical procedures and losses in the LDS layer.

Heavy metal cations, such as Cd^2+^, Pb^2+^, and Hg^2+^, have been widely used to synthesize QDs due to their remarkable optical characteristics and tunable PL emission. Nevertheless, disposability, toxicity, and other long-lasting health hazards associated with the use of these heavy-metal compounds are increasingly important issues. Non-cadmium substitutes with adequate stability have been explored, and it has been suggested that these are essential requirements for research and development. Consequently, substantial effort has been made to produce heavy-metal-free (HMF) QDs, besides a wide variety of compounds and fundamental semiconductors that are less hazardous to the environment. To minimize the cost of QD production and device fabrication, convenient methods with a decent price, harmless precursors, affordable and environmentally sustainable solvents, cost-effective processing techniques, low-energy synthetic approaches, and infrastructure are required. In terms of device fabrication, the issue of significant performance loss in a variety of QD devices caused by fabricating devices in an atmospheric environment must be addressed. To facilitate their implementation in solar panels, more attention should be focused on the manufacture of uniform and large-area QD films.

## 7. Conclusions

In summary, the possibilities for a more appropriate implementation of the short wavelength of light (especially in the UV region) via the LDS of solar irradiance have been explained and reviewed in detail. CQDs and PQDs were both found to be good alternatives to enhance the response in the UV region for any kind of solar cell compared to other luminescent materials, due to their unique properties, such as their tunable absorption and emission band, near-unity quantum yields, and simplistic synthesis processes. Furthermore, different synthesis methods and stability improvement strategies for QDs were discussed extensively. In the same vein, the recent breakthrough in Si, GaAs, CdTe, CIGS, organic, and perovskite solar cells with QD LDS layers was addressed at length. This review can provide a brief overview of the past, present, and future developments of QD-LDS-layer-based solar cells. Improvements in the synthesis techniques for QDs and the device fabrication for a solar cells could lead to a more viable technology for next-generation solar cells. Further study into the stability and protection approaches of QD coatings on solar cells is necessary to meet the demanding stability requirements for commercial applications. Although researchers have made remarkable improvements in the stability and PCE of solar cells when combined with QDs, further development is still needed to achieve a level of stability and PCE that is suitable for commercialization. We expect that this review will provide an efficient route to QD-LDS-layer-based solar cells and the future demands of next-generation solar cells.

## Figures and Tables

**Table 1:** Various synthesis methods of QDs in terms of PLQY improvement.

QD Type	Synthesis Method	PLQY (%)	Ref./Year
CdSe–ZnS	Microwave	50	[121]/2008
CH_3_NH_3_PbBr_3_	Ligand-assisted reprecipitation (LARP)	70	[110]/2015
CsPbBr_3_	Hot injection and RT-SR	90	[111]/2016
Ligand-capped CdS	Solvothermal	69	[99]/2016
CH_3_NH_3_PbBr_3_	Spray synthesis	100	[116]/2018
CsPbBr_3_	Room-temperature triple-ligand surface engineering	93	[112]/2018
CdSe/ZnS core/shell	Hydrothermal	47	[106]/2019
MAPbBr_3_/PQDP	Oleic acid/oleylamine (traditional surface ligands)-free	80	[120]/2020
CH_3_NH_3_Pb_1−x_Co_x_Br_3−2x_Cl_2x_	Cation and anion exchange	95	[119]/2021

**Table 2:** List of reported work on QD LDS layers for silicon solar cells.

Si Type	QD Type	Matrix	Enhancement in J_sc_ (%)	Enhancement in PCE (%)	Ref./Year
c-Si	CdS	Silica	4	N/A	[43]/2010
c-Si	CdS	N/A	2.76	33	[32]/2011
N/A	ZnSe	SiO_2_	23.75	12.88	[164]/2012
N/A	CdSe/ZnS	N/A	6.21	5.5	[165]/2014
c-Si	CdSe/ZnS	N/A	6.55	3.58	[166]/2014
N/A	CdZnS/ZnS	N/A	6.45	6.4	[167]/2014
N/A	CuInS_2_/ZnS	N/A	28	37.5	[168]/2014
c-Si	CdSe/CdS/ZnS	SiO_2_	2.61	5.2	[169]/2015
N/A	CuInS_2_/ZnS	PMMA	2.78	3.95	[170]/2015
N/A	CH_3_NH_3_PbBr_3_	PVA ^&^	12.96 (I_sc_)	45	[171]/2015
c-Si	CdSe/ZnS/Ag NPs	PMMA	7.84	5.95	[172]/2016
mc-Si	CsPbCl_3_:Mn^2+^	N/A	5.1	6.2	[162]/2018
N/A	ZnSe/ZnS/Ag NPs	N/A	12.5	N/A	[173]/2018
p-Si	CdTe	PMMA	17.39 (I_sc_)	48.47	[158]/2018
c-si	CH_3_NH_3_PbBr_3_	PAN ^$^	4.03	6.69	[17]/2019
mc-Si	CH_3_NH_3_PbBr_3_	PAN ^$^	2.07	8.43	[17]/2019
IBC *	CdSe_1−x_S_x_/ZnS	N/A	39.5	40	[174]/2019
a-Si	Cd_x_Zn_1−x_Se_y_S_1−y_/ZnS	PDMS	9.7 (I_sc_)	11.73	[175]/2020
c-Si	CuInS_2_/ZnS/ZnS	EVA ^#^	1.8 (I_sc_)	2.1	[176]/2020

^&^ polyvinyl alcohol, ^$^ polyacrylonitrile, * interdigitated back contact, ^#^ ethylene−vinyl acetate.

**Table 3:** List of reported work on QD LDS layers for GaAs solar cells.

QD Type	Matrix	Enhancement in J_sc_ (%)	Enhancement in PCE (%)	Ref./Year
CdS	PDMS	18.37	21.52	[40]/2012
CdS	N/A	20.98	18.91	[177]/2012
CdSe	N/A	9.78	10.4	[182]/2013
CdSe/ZnS	N/A	21.9	24.65	[33]/2014
CdZnS/ZnS	PDMS	3.36	3.24	[183]/2015
CdSe/ZnS	InvisiSil	N/A	2.87	[18]/2015
CdS/ZnS	N/A	4.43	3.77	[184]/2016
CdS/ZnS	N/A	9.44	5.78	[184]/2016
CdS/ZnS	N/A	12.24	17.64	[185]/2017
CdSe/ZnS	N/A	44.6	5.47	[59]/2018
CsPbBr_3_	N/A	10.9	14.06	[178]/2018
CsPbBr_3_	PDMS/SiO_2_	5.22	7.12	[41]/2018
CsPbBr_3_	PDMS/SiO_2_	3.60	10.56	[180]/2020
FAPbBr_3_	N/A	18	N/A	[181]/2021

**Table 4:** List of reported work on QD LDS layers for CdTe solar cells.

QD Type	Matrix	Enhancement in J_sc_ (%)	Enhancement in PCE (%)	Ref./Year
CdS	PMMA	30.3	1.7	[188]/2013
Commercial	PMMA	4	N/A	[189]/2013

**Table 5:** List of reported work on QD LDS layers for CIGS solar cells.

QD Type	Matrix	Enhancement in J_sc_ (%)	Enhancement in PCE (%)	Ref./Year
CdS/ZnS	N/A	12.2	10.9	[191]/2015
CdSe/CdZnS	N/A	4.35	3.85	[192]/2017
CdSe	PMMA	9.54	N/A	[35]/2019
CdSe/CdZnS	N/A	7.33	7.40	[19]/2019
CsPbBr_3_	N/A	4.5	4.5	[193]/2020

**Table 6:** List of reported work on QD LDS layers for organic and PSC solar cells.

Cell Type	QD Type	Position of LDS Layer	Enhancement in J_sc_ (%)	Enhancement in PCE (%)	Ref./Year
Organic	CuGaSe_2_	Inside	9.43	23.8	[200]/2012
PSCs	ZnSe	Outside	0.5	4.21	[196]/2017
PSCs	CsPbCl_3_:Mn	Outside	3.77	3.34	[201]/2017
PSCs	CdSe	Inside	8.06	14.67	[202]/2017
QDSCs	CsPbBr_3_	Inside	−4.84 (V_oc_ enhancement: 23.40%)	50.41	[203]/2017
PSCs	CuInS_2_	Inside	18.12	31.46	[204]/2018
PSCs	CdSe	Inside	12.03	35.5	[205]/2018
PSCs	Cs^3+^-CsPbI_3_	Outside	2.9	3.06	[198]/2019
PSCs	CsPbBr_3_	Inside	3.64	11.6	[199]/2019
PSCs	CsPbBr_3_@SiO_2_	Outside	4.53	5.6	[199]/2019

## Data Availability

Not applicable.
